# Mammalian Cytochrome P450-Dependent Metabolism of Polychlorinated Dibenzo-*p*-dioxins and Coplanar Polychlorinated Biphenyls

**DOI:** 10.3390/ijms150814044

**Published:** 2014-08-13

**Authors:** Hideyuki Inui, Toshimasa Itoh, Keiko Yamamoto, Shin-Ichi Ikushiro, Toshiyuki Sakaki

**Affiliations:** 1 Research Center for Environmental Genomics, Kobe University, 1-1 Rokkodaicho, Nada-ku, Kobe, Hyogo 657-8501, Japan; 2 Laboratory of Drug Design and Medicinal Chemistry, Showa Pharmaceutical University, 3-3165 Higashi-tamagawagakuen, Machida, Tokyo 194-8543, Japan; E-Mails: titoh@ac.shoyaku.ac.jp (T.I.); yamamoto@ac.shoyaku.ac.jp (K.Y.); 3 Biotechnology Research Center, Faculty of Engineering, Toyama Prefectural University, 5180 Kurokawa, Imizu, Toyama 939-0398, Japan; E-Mails: ikushiro@pu-toyama.ac.jp (S.-I.I.); tsakaki@pu-toyama.ac.jp (T.S.)

**Keywords:** CYP1A1, cytochrome P450 monooxygenase, hydroxylation, polychlorinated biphenyl, polychlorinated dibenzo-*p*-dioxin, sulfotransferase, UDP-glucuronosyltransferase

## Abstract

Polychlorinated dibenzo-*p*-dioxins (PCDDs) and coplanar polychlorinated biphenyls (PCBs) contribute to dioxin toxicity in humans and wildlife after bioaccumulation through the food chain from the environment. The authors examined human and rat cytochrome P450 (CYP)-dependent metabolism of PCDDs and PCBs. A number of human CYP isoforms belonging to the CYP1 and CYP2 families showed remarkable activities toward low-chlorinated PCDDs. In particular, human CYP1A1, CYP1A2, and CYP1B1 showed high activities toward monoCDDs, diCDDs, and triCDDs but no detectable activity toward 2,3,7,8-tetrachlorodibenzo-*p*-dioxin (2,3,7,8-tetraCDD). Large amino acids located at putative substrate-recognition sites and the F-G loop in rat CYP1A1 contributed to the successful metabolism of 2,3,7,8-tetraCDD. Rat, but not human, CYP1A1 metabolized 3,3',4,4',5-pentachlorobiphenyl (CB126) to two hydroxylated metabolites. These metabolites are probably less toxic than is CB126, due to their higher solubility. Homology models of human and rat CYP1A1s and CB126 docking studies indicated that two amino acid differences in the CB126-binding cavity were important for CB126 metabolism. In this review, the importance of CYPs in the metabolism of dioxins and PCBs in mammals and the species-based differences between humans and rats are described. In addition, the authors reveal the molecular mechanism behind the binding modes of dioxins and PCBs in the heme pocket of CYPs.

## 1. Introduction

**Figure 1:**
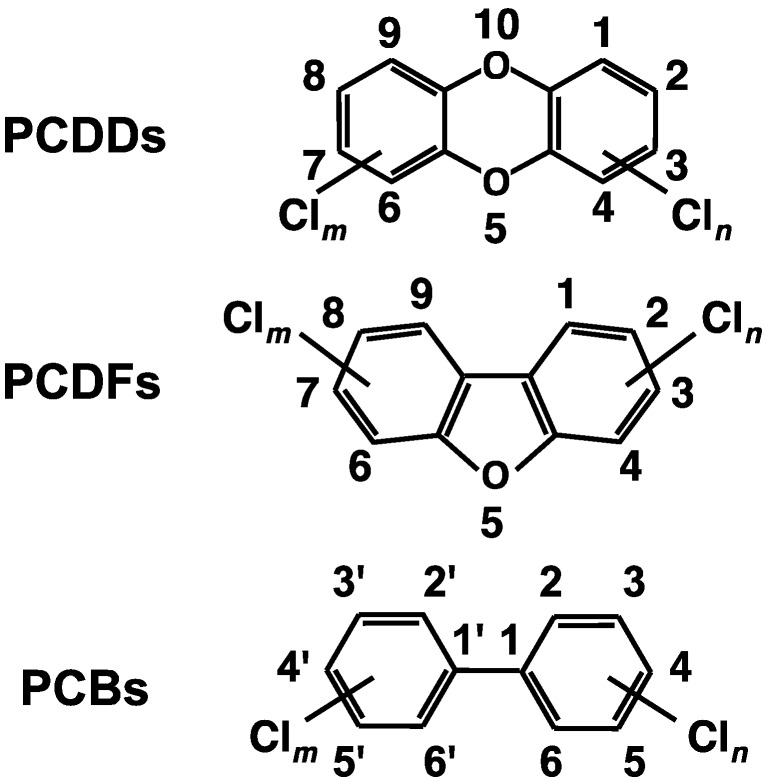
Structures of polychlorinated dibenzo-*p*-dioxins (PCDDs), polychlorinated dibenzofurans (PCDFs), and polychlorinated biphenyls (PCBs).

Dioxins containing polychlorinated dibenzo-*p*-dioxins (PCDDs) and polychlorinated dibenzofurans (PCDFs) are generated naturally through processes such as forest fires and waste incineration; dioxins also are generated as byproducts of industrial processes (Figure 1). In contrast, coplanar polychlorinated biphenyls (PCBs) have been produced commercially (Figure 1). They are considered environmental contaminants because of the extreme toxicity of some family members, with 2,3,7,8-tetrachlorodibenzo-*p*-dioxin (tetraCDD) being considered the most toxic. The World Health Organization (WHO) recommends a maximum total daily intake of 1–4 pg I-TEQ/kg body weight [1]. Human exposure to dioxins appears to come predominantly from meat, fish, and dairy products [2]. Human breast milk can also be contaminated with dioxins [3].

Toxicity evaluations of chemicals are carried out by using experimental animals, such as mice, rats, and dogs, because humans are not exposed to chemicals, the safety of which is unclear. Safety data obtained from experimental animals are extrapolated to humans using safety factors. Usually, a safety factor of 100 is applied to the No Observed Adverse Effect Level (NOAEL) dose derived from animal experiments. This figure is a product of factors for inter-species (10) and intra-species (10) differences. These factors are important to take into account when considering safety differences between experimental animals and humans; however, there are often large differences between mammals in terms of their sensitivity toward chemicals.

**Figure 2:**
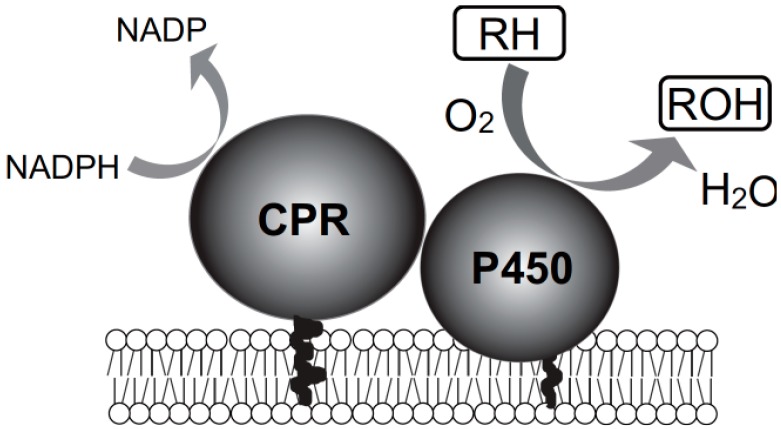
Cytochrome P450 (P450, CYP) monooxygenases. RH, substrate; and CPR, NADPH-P450 oxidoreductase.

The metabolism of PCDDs has been studied *in vivo* by using experimental animals [4,5,6,7,8,9,10], and *in vitro* by using liver slices and liver microsomal fractions [9]. The major metabolites are hydroxylated products, glucuronide conjugates, and sulfate conjugates [5,7]. PCDD metabolism involves the initial insertion of a single oxygen atom into the PCDD molecule to form an epoxide, probably by cytochrome P450 (CYP)-dependent monooxygenases (Figure 2). Hu and Bunce [9] studied the metabolism of PCDDs in microsomal fractions prepared from 3-methylcholanthrene (MC)-treated rats and suggested that CYP1A1 and CYP1A2 play an important role in the metabolism of PCDDs. Their findings strongly suggest that CYP-dependent monooxygenases are key enzymes for the metabolism of PCDDs and that phase II enzymes, including UDP-glucuronosyltransferase (UGT) and sulfotransferase (SULT), also play important roles in the metabolism of PCDDs.

Ten years ago, Ukrainian President Victor Yushchenko experienced severe 2,3,7,8-tetraCDD poisoning. The 2,3,7,8-tetraCDD level in his blood serum was 108,000 pg/g lipid weight, which was more than 50,000-fold higher than the levels in the general population. Two metabolites of 2,3,7,8-tetraCDD were detected in his feces [11]; however, there were no published reports identifying the human CYP isoforms responsible for this 2,3,7,8-tetraCDD metabolism, although CYP1A1, CYP1A2, and CYP1B1 are known to be induced by 2,3,7,8-tetraCDD in humans [12,13].

PCBs were once widely used as materials for industrial products such as transformers and condensers because of their characteristic non-flammability, low electrical conductivity, and chemical stability. The PCBs in materials are a mixture of chlorinated biphenyls containing one to 10 chlorine atoms on the biphenyl rings, and there are 209 isoforms, which are termed congeners. Among these congeners, coplanar PCBs, which are non-*ortho* and mono-*ortho*, exhibit a dioxin-like toxicity toward mammals [14]. Coplanar PCBs have a planar structure similar to that of dioxins and bind aryl hydrocarbon (AhR) receptors, resulting in the transcriptional activation of genes responsible for the expression of dioxin-like toxicity [15]. Although PCBs have been banned in most (if not all) industrialized countries, not just Japan, PCBs have continued to cause environmental pollution due to their leakage from products and their persistency in the environment [16]. Environmental contamination by PCBs has been detected even in the polar regions as well as in industrial areas due to the ability of PCBs to migrate long distances. The PCBs bio-accumulate in the adipose tissues of wildlife via the food chain, with high accumulation leading to dioxin-like toxicity [17].

CYP-dependent monooxygenases are also involved in the metabolism of some PCBs. MC-treated rats that received 3,3',4,4',5-pentaCB (CB126), which is the most toxic of the PCB congeners, metabolized CB126 to 4-OH-3,3',4',5,5'-pentaCB, which was detected in their feces [18]. Haraguchi *et al.* detected five different metabolites, including 4- and 5-hydroxy metabolites, from rats treated with CB126 [19]. Rat, but not human, CYP1A1 metabolized CB126 to hydroxylated metabolites [20]. This *in vitro* experiment revealed that CYPs were responsible for the hydroxylation of CB126. In rats, hamsters, and guinea pigs treated with the CYP inducers phenobarbital (PB) and MC, 2,2',3,4',5,5',6-heptaCB (CB187) was metabolized to different hydroxylated metabolites depending on the animal species [21]. These results showed that there are species-specific differences with regard to the production of metabolites, indicating that the expression levels of *CYP* genes and the activities of the gene products differ among mammals.

## 2. Yeast Expression System for Mammalian Cytochrome P450 (CYP) Isoforms

Three decades ago, heterologous expression of mammalian *CYP* genes using a *Saccharomyces cerevisiae* expression system succeeded [22]. The expressed rat CYP1A1 was localized on the yeast endoplasmic reticulum membrane and received electrons from yeast NADPH–P450 oxidoreductase (reductase) to demonstrate monooxygenase activity [23]. Yeast cells appear to have a machinery similar to that of mammalian cells with regard to the co-translational localization of mammalian microsomal CYPs. Although the *S. cerevisiae* genome contains three microsomal *CYP* genes [24], the gene products have no detectable activity toward xenobiotics, such as drugs, dioxins, and PCBs. *In vitro* studies revealed that the efficiency of electron transfer from yeast reductase to mammalian CYPs is nearly the same as that from mammalian reductase [25]. Human *CYP1A1*, *CYP1A2*, *CYP1B1*, *CYP2A6*, *CYP2B6*, *CYP2C8*, *CYP2C9*, *CYP2C18*, *CYP2C19*, *CYP2D6*, *CYP2E1*, and *CYP3A4* genes have successfully been expressed in *S. cerevisiae* [26,27]. This system is thus useful for identifying CYPs involved in drug metabolism. One of the advantages of the yeast expression system is that whole cells of recombinant *S. cereviasiae* producing human CYPs can be used as biocatalysts for the biosynthesis of metabolites. This system was used to predict the metabolism of PCDDs and coplanar PCBs in humans.

## 3. Metabolism of Polychlorinated Dibenzo-*p*-dioxins (PCDDs) by Human and Rat CYPs

### 3.1. Metabolism of 2,3,7,8-Tetrachlorodibenzo-p-dioxin (tetraCDD) in Humans

As mentioned in the Introduction, in 2004, Ukrainian President Victor Yushchenko experienced severe 2,3,7,8-tetraCDD poisoning. The 2,3,7,8-tetraCDD level in his blood serum was 108,000 pg/g lipid weight (50,000-fold above the normal level). Sorg *et al.* [11] monitored the levels of 2,3,7,8-tetraCDD and its metabolites in blood serum, subcutaneous fat, feces, sweat, and urine for 3 years; 8-OH-2,3,7-triCDD and 2-OH-1,3,7,8-tetraCDD were detected as metabolites in the feces, and the amount of the former was approximately two times higher than that of the latter. Trace amounts of these metabolites were detected in the serum and urine. The half-life of 2,3,7,8-tetraCDD in Victor Yushchenko’s body was estimated to be 15.4 months. Of note, these two metabolites of 2,3,7,8-tetraCDD have also been detected in dogs [6]. The authors believe that CYPs were responsible for the hydroxylation of 2,3,7,8-tetraCDD for the following reasons: (1) The contributions of other enzymes (*i.e.*, not CYPs) to the oxidation of PCDDs may be excluded [28]; (2) Spectral analysis strongly suggested that human CYP1A1 binds 2,3,7,8-tetraCDD in its substrate-binding pocket [29]; and (3) Human recombinant CYP1A2-dependent activity was inhibited by 2,3,7,8-tetraCDD [30].

**Figure 3:**
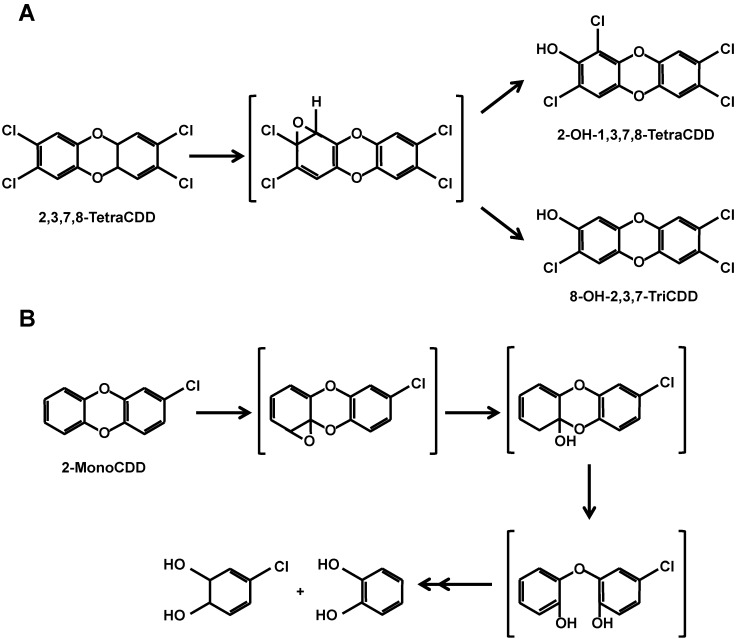
Metabolic pathways for 2,3,7,8-tetraCDD (**A**) and 2-monoCDD (**B**) involving CYPs.

Furthermore, the authors concluded that CYPs belonging to the CYP1 family, that is, CYP1A1, CYP1A2, and/or CYP1B1, could metabolize 2,3,7,8-tetraCDD, although their activity was too subtle to detect under *in vitro* procedures. The metabolites 8-OH-2,3,7-triCDD and 2-OH-1,3,7,8-tetraCDD that were detected in Victor Yushchenko were likely produced by CYPs belonging to the CYP1 family whose expression was induced by 2,3,7,8-tetraCDD (Figure 3A) [31].

### 3.2. CYP-Dependent Metabolism of PCDDs

Recombinant yeast microsomal fractions containing all the human CYPs were examined for their participation in the metabolism of PCDDs. Multiple CYP isoforms showed remarkable metabolic processes toward dibenzo-*p*-dioxin (DD), 1-monoCDD, 2-monoCDD, 2,3-diCDD, 2,7-diCDD, and 2,3,7-triCDD [29] (Figure 3B). These metabolic processes included multiple reactions such as hydroxylation at an unsubstituted position, hydroxylation with migration of a chlorine substituent, and hydroxylation with elimination of a chlorine substituent. Clear differences were observed among the CYP1, CYP2, and CYP3 families. The CYP1 family showed high activities toward DD and mono-, di-, and triCDDs and remarkable activities toward 2,7-diCDD and 2,3,7-triCDD. In contrast, the CYP2 family showed activities toward DD, 1-monoCDD, 2-monoCDD, and 2,3-diCDD but no activities toward 2,7-diCDD and 2,3,7-triCDD. CYP3A4, which is the most important CYP in drug metabolism, showed no activities toward PCDDs. None of the CYPs showed detectable activity toward 2,3,7,8-tetraCDD.

### 3.3. Binding of Tetra- and PentaCDDs to the Substrate-Binding Pocket of CYPs

As described earlier, none of the CYPs showed activity toward 2,3,7,8-tetraCDD [29]. So, can CYPs bind 2,3,7,8-tetraCDD or not? To answer this question, tetraCDD-induced difference spectra were measured. Addition of 2,3,7-triCDD or 2,3,7,8-tetraCDD to microsomal fractions containing human CYP1A1 induced typical type I spectra, indicating a change in the heme iron of CYP1A1 from a low-spin state to a high-spin state upon binding of 2,3,7-triCDD or 2,3,7,8-tetraCDD. The value of the dissociation constant *K*_d_ for 2,3,7-triCDD was estimated to be 0.65 µM. Although the authors did not determine the *K*_d_ value for 2,3,7,8-tetraCDD, the affinity for 2,3,7,8-tetraCDD appeared to be not particularly different from that for 2,3,7-triCDD [30]. These results indicate that CYP1A1 can bind 2,3,7,8-tetraCDD in its substrate-binding pocket but shows no detectable activity toward it.

Staskal *et al.* reported that human recombinant CYP1A2-dependent activity was inhibited by 2,3,7,8-tetraCDD, 1,2,3,7,8-pentaCDD, 2,3,7,8-tetraCDF, and 2,3,4,7,8-pentaCDF, with *K*_i_ values of less than 1 μM [30]. These results indicate that CYP1A2 can bind to these dioxins with high affinity. On the basis of these results, PCDDs and PCDFs with five chlorine substituents could dock into the substrate-binding pocket of CYPs belonging to the CYP1 family.

### 3.4. Further Metabolism of PCDDs after CYP-Dependent Hydroxylation

*In vivo* studies using experimental animals have demonstrated that the major metabolites of PCDDs are hydroxylated products, glucuronide conjugates, and sulfate conjugates [5,7]. The formation of glucuronides or sulfates is an indispensable step in the detoxification of lipophilic compounds, which subsequently are transformed into more hydrophilic metabolites and excreted in bile or urine. Glucuronidation of steroids, bile acids, bilirubin, hormones, drugs, and environmental toxicants is catalyzed by UDP glucuronosyltransferases (UGTs), whose gene family in the human genome contains 19 species with different substrate specificities [32]. No human UGT species that catalyzes the glucuronidation of PCDDs has yet been identified, although some human UGT species appear to be involved in the metabolism of PCDDs.

### 3.5. Metabolism of 8-OH-2,3,7-triCDD by Human Recombinant UDP Glucuronosyltransferases (UGTs)

The additional metabolism of dioxins by UGTs that occurs after the CYP-dependent reaction was examined. 8-OH-2,3,7-triCDD, a major metabolite of 2,3,7-triCDD formed via the activity of CYP1A1, CYP1A2, and CYP1B1, served as a substrate for UGT [33]. Because 8-OH-2,3,7-triCDD is a major metabolite of 2,3,7,8-tetraCDD in mammals [6,34], it is important to understand the metabolism of 8-OH-2,3,7-triCDD to fully understand the metabolism of 2,3,7,8-tetraCDD in mammals. The metabolism of 8-OH-2,3,7-triCDD by 12 species of human UGTs was examined by using recombinant UGTs expressed in baculovirus-infected insect cells (Supersomes, BD Sciences, San Jose, CA, USA). Surprisingly, 2,3,7-triCDD glucuronide was detected in the recombinant system containing each of UGT1A1, UGT1A3, UGT1A7, UGT1A8, UGT1A9, UGT1A10, UGT2B7, UGT2B15, and UGT2B17, yet it was not detected in systems containing each of UGT1A4, UGT1A6, and UGT2B4. Thus, 8-OH-2,3,7-triCDD is a good substrate for most human UGT species.

### 3.6. Successive Metabolism of 2,3,7-triCDD by CYPs and UGTs in Human Liver Microsomes

As described in the previous sections, heterologous expression systems for CYPs and UGTs are very useful for predicting the metabolism of dioxins. In addition, commercially available human liver microsomes are also useful for understanding the metabolism of dioxins in the human body. CYP-dependent 2,3,7-triCDD hydroxylation activities in human liver microsomes from 10 individual human livers in the presence of NADPH were measured [33]. A significant difference in hydroxylation activity was observed among the different microsomal preparations from the 10 human livers, and the activity range was 15-fold. Although both human CYP1A1 and CYP1B1 have high 2,3,7-triCDD 8-hydroxylation activity, their expression levels in human liver are quite low, whereas CYP1A2 is a major CYP isoform in the liver [35]. Therefore, the authors predicted that CYP1A2 was the major CYP that catalyzed the hydroxylation of 2,3,7-triCDD in human liver. Because phenacetin *O*-deethylation is specifically metabolized by CYP1A2 [36], the authors examined the correlation between 2,3,7-triCDD 8-hydroxylation and phenacetin *O*-deethylation in human liver microsomes. As expected, a good correlation (*r* = 0.92) was observed between the two reactions. These results strongly suggest that CYP1A2 is responsible for 2,3,7-triCDD 8-hydroxylation in human liver, and the significant difference in the activity among the microsomal preparations from the 10 different human livers likely reflected differences in the CYP1A2 contents of the liver microsomes. In contrast, glucuronidation activity toward 8-OH-2,3,7-triCDD was not significantly different among the different microsomal preparations from the 10 human livers; the activity range was only 1.9-fold. A time course of 2,3,7-triCDD metabolisms on human liver microsomes in the presence of NADPH and UDP-glucuronic acid was also examined [33]. Initially, 8-OH-2,3,7-triCDD levels increased linearly with time, and then reached a plateau. Glucuronide formation was detected, but after 8-OH-2,3,7-triCDD accumulation following a lag phase, glucuronide levels then increased linearly with time. The time courses of these metabolites thus represent the typical sequential conversion by a two-enzyme system.

### 3.7. Species-Based Difference in CYP-Dependent Metabolism of PCDDs between Humans and Rats

Human and rat CYP1A-dependent metabolism of PCDDs by CYP1A subfamily members by using recombinant yeast microsomes was compared. Considerable species differences between humans and rats were observed for both CYP1A1- and CYP1A2-dependent metabolism of dioxins. Among four CYPs, rat CYP1A1 showed the highest activity toward DD, mono-, di-, and triCDDs [37].

### 3.8. Generation of 2,3,7,8-tetraCDD-Metabolizing CYPs by Modifying Rat CYP1A1 through Site-Directed Mutagenesis

**Figure 4:**
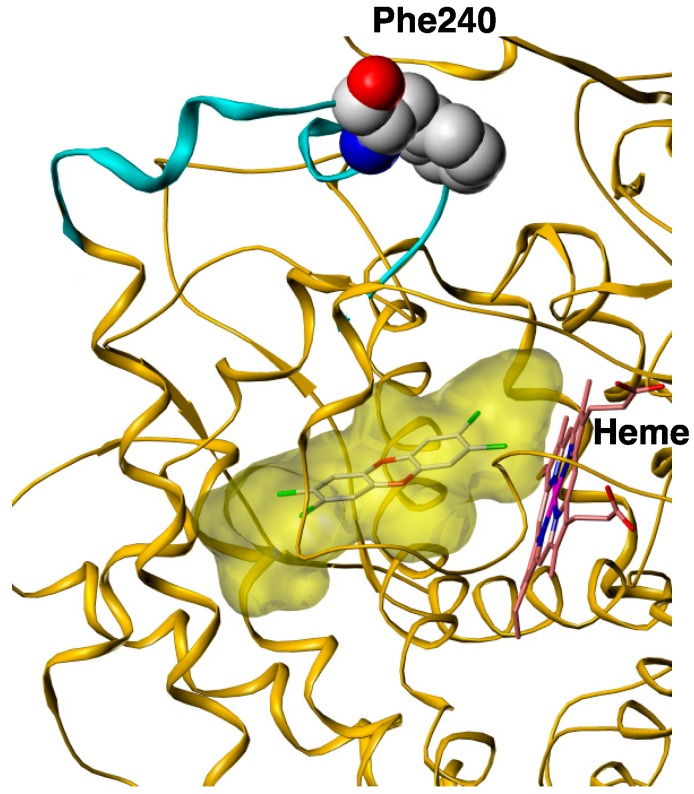
A docking model of 2,3,7,8-tetraCDD into rat CYP1A1. The cyan ribbon represents the F–G loop. The yellow shaded region is the substrate-binding cavity.

The extremely high toxicity of 2,3,7,8-tetraCDD derives from its high affinity for the Ah receptor and its nearly undetectable metabolism in the mammalian body [9]. Given that 2,3,7-triCDD is a good substrate for rat CYP1A1, the authors hypothesized that enlargement of the space for the putative substrate-binding pocket and of the substrate access channel of rat CYP1A1 might generate catalytic activity toward 2,3,7,8-tetraCDD. Large amino acid residues such as Phe, Tyr, Leu, and Ile that are involved in substrate binding and substrate entry were substituted for alanine by using site-directed mutagenesis. Among eight mutants examined, the mutant in the putative F–G loop, F240A, catalyzed the conversion of 2,3,7,8-tetraCDD to 8-OH-2,3,7-triCDD [38]. Because the affinity of 8-OH-2,3,7-triCDD for Ah receptor was less than 0.001% of that for 2,3,7,8-tetraCDD, this metabolic event resulted in remarkable detoxification of 2,3,7,8-tetraCDD. A docking model of 2,3,7,8-tetraCDD with rat CYP1A1 on the basis of the crystal structure of human CYP1A2 was constructed [20,39,40] using homology modeling. Figure 4 shows the docking model of 2,3,7,8-tetraCDD in the active site of rat CYP1A1. 2,3,7,8-tetraCDD is accommodated in the planar active site. This model concurs well with the experimental result that rat CYP1A1 can bind 2,3,7,8-tetraCDD as described in Section 3.2. The figure shows the three-dimensional overall structure of rat CYP1A1 Phe240 located in the F–G loop, which is associated with the membrane and involved in substrate entry. As mentioned in the Section 3.1, 8-OH-2,3,7-triCDD is a major metabolite of 2,3,7,8-tetraCDD in mammals [6,34]. These *in vivo* studies thus appear to be inconsistent with our results showing that native CYPs have no catalytic activity toward 2,3,7,8-tetraCDD. However, it is possible that native CYP1A may be able to convert 2,3,7,8-tetraCDD to 8-OH-2,3,7-triCDD.

## 4. Metabolism of PCBs by Human and Rat CYPs

### 4.1. In Vitro Metabolism of CB126 with Microsomal Fractions from Recombinant Yeast

Microsomal fractions containing human and rat CYP1A1s were prepared from recombinant *S. cerevisiae* expressing the corresponding genes [22]. With NADPH as an electron donor and the NADPH-recycling system using gluose-6-phosphate and glucose-6-phosphate dehydrogenase, CB126 was mixed with the microsomal fractions. After a 2-h incubation at 37 °C with shaking, ^13^C-hydroxylated PCBs, as internal standards, were added to the reaction mixture. CB126 and its metabolites were extracted with hexane from the reaction mixture and subjected to methylation [41]. The residues dissolved in hexane were subjected to high-resolution gas chromatography–high-resolution mass spectrometry. Rat CYP1A1 showed two NADPH-dependent peaks, whereas no peaks were detected with human CYP1A1 [20]. The vector control also showed no detectable peaks.

### 4.2. Identification of CB126 Metabolites

Koga *et al*. reported that rats that had orally been administered CB126 excreted 4-OH-3,3',4',5,5'-pentaCB as a major metabolite in their feces [18]. However, the CYP isoforms involved in this reaction were not identified. The authors showed that rat CYP1A1 produced two hydroxylated metabolites, identified as 4-OH-3,3',4',5-tetraCB and 4-OH-3,3',4',5,5'-pentaCB on the basis of the retention times of the authentic standards, their isotope ratios, and their fragment ion patterns [20]. Much more of the newly identified metabolite 4-OH-3,3',4',5-tetraCB than of 4-OH-3,3',4',5,5'-pentaCB was produced *in vitro*. These results suggest that CB126 is detoxified by rat CYP1A1 because PCB congeners that are less chlorinated and hydroxylated generally have less hydrophobicity (*i.e*., increased body clearance) and decreased binding affinity for the Ah receptor that is responsible for dioxin toxicity [18]. In contrast, human CYP1A1 did not produce detectable metabolites, and rat CYP1A1 in the reaction mixture without NADPH did not yield either peak for the hydroxylated metabolites. These results strongly suggest that CB126 is more toxic for humans than for rats due to its bioaccumulation in humans. Dioxin toxicity, including PCB toxicity, has been defined by estimations obtained largely from *in vivo* experiments using animals. The species-specific differences between humans and rats in terms of PCB metabolism could lead to misinterpretation of toxicity evaluations.

### 4.3. Molecular Modeling of Human and Rat CYP1A1s

**Figure 5:**
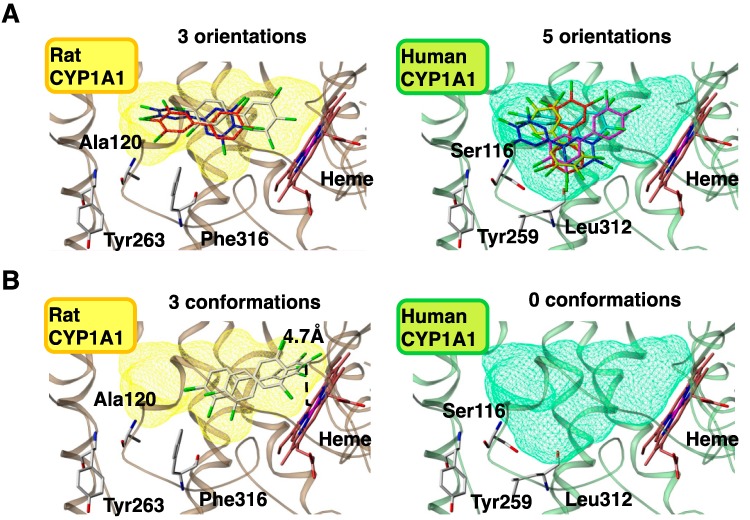
Docking models of CB126 into rat and human CYP1A1s. Orientation numbers of CB126 in a substrate-binding cavity (**A**) and conformation numbers of CB126 within 5 Å of the heme (**B**). Left and right panels show rat and human CYP1A1s, respectively. Yellow and green shades indicate substrate-binding cavities of rat and human CYP1A1s, respectively.

Human CYP1A1 shares 79% amino acid sequence homology with rat CYP1A1. Four amino acid residues—Ser116, Ser122, Asn221, and Leu312 for humans and the corresponding amino acids Ala120, Thr126, Ser225, and Phe316 for rats—are not conserved among the amino acid residues that comprise the substrate-binding cavity (Table 1). These residues contribute to the differences in the metabolism of CB126. To clarify the influence of these amino acid differences on metabolic activities, 3D structures of human and rat CYP1A1s were constructed. The 3D structure of human CYP1A1 was based on human CYP1A2 [39], and that of rat CYP1A1 was based on the constructed human CYP1A1 [40]. In the rat CYP1A1 model, there was a steric crush error between Tyr263 on the G-Helix and Phe316 on the I-Helix, whereas no errors occurred in the human CYP1A1 model (Figure 5). Therefore, it was thought that the side chain of Phe316 in rat CYP1A1 flips into the substrate-binding cavity given that Ala120 is sufficiently small enough to allow the flip. This creates a smaller cavity volume (510 Å) compared with that of human CYP1A1 (600 Å). Leu312 in human CYP1A1 does not conflict with Tyr259, corresponding to Tyr263 in rat CYP1A1, because the side chain of Leu is smaller than that of Phe. Leu is mostly conserved in animals, including dog, guinea pig, monkey, mouse, and rabbit, although the rat has Phe (Table 1). Furthermore, Ala120 in rat CYP1A1 is unique among these animals. The combination of Ala120 and Phe316 confers unique metabolic activities to rat CYP1A1 by decreasing the cavity volume.

**Table 1:** Structures of the amino acid residues in the substrate-binding pocket in mammalian CYP1A1s.

Rat	Human	Dog	Golden Hamster	Guinea Pig	Monkey	Mouse	Rabbit
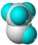	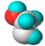	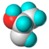	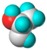	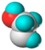	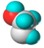	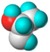	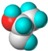
**Ala**	**Ser**	**Thr**	**Thr**	**Ser**	**Ser**	**Thr**	**Thr**
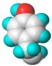	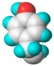	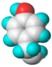	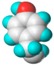	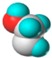	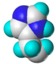	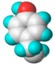	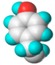
**Tyr**	**Tyr**	**Tyr**	**Tyr**	**Ser**	**His**	**Tyr**	**Tyr**
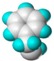	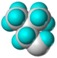	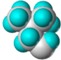	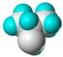	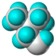	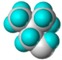	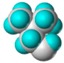	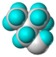
**Phe**	**Leu**	**Leu**	**Val**	**Leu**	**Leu**	**Leu**	**Leu**

Mammalian amino acids corresponding to Ala120, Tyr263, and Phe316 of rat CYP1A1 are represented.

### 4.4. Construction of Docking Models with CB126 and CYP1A1s

Docking models revealed that CB126 was more stable in the cavity of rat CYP1A1 than in that of human CYP1A1 [20] (Figure 5A). This stability was due to less orientation of CB126 in the cavity of rat CYP1A1 relative to that in human CYP1A1. In contrast, CB126 was more accessible to the heme of rat CYP1A1 than to that of human CYP1A1 (Figure 5B); three conformations were predicted to be close to the heme (less than 5 Å) of rat CYP1A1, whereas no conformation was in human CYP1A1. Less than 5 Å between the four-position of the carbon in CB126 and the iron of the heme in CYPs is necessary for the reaction to occur. These results reveal that the orientation of PCBs in the cavity of CYPs and the ability to be close to the iron of the heme of CYP1A1 stem from the differences in the CYP amino acids in different species and their subsequent effects on PCB metabolism.

## 5. Conclusions

Here, the authors described the metabolism of PCDDs and PCBs via CYP-dependent hydroxylation. Molecular modeling of CYPs and the construction of docking models with these compounds explained the reaction mechanisms based on differences in amino acid sequence. Appropriate mutations at these amino acids made it possible to create novel CYPs with higher metabolic activity. This approach can be applied to the efficient remediation of PCDDs and PCBs. It is important to understand the metabolism of these compounds with respect to their toxicity for humans, but it is unacceptable to administer these compounds to humans. Therefore, toxicity data from experimental animals are extrapolated to estimate toxicity toward humans. However, there are metabolic differences between experimental animals and humans, which complicate such toxicity determinations. Our approach to *in vitro* CYP-dependent metabolism of PCDDs and PCBs is a promising method to more accurately evaluate toxicity for humans.
